# IMPROVEMENT IN FINE MOTOR CONTROL IN DEMENTIA ELDERLY FROM A COMPUTERIZED TOUCHSCREEN TRAINING PROGRAM

**DOI:** 10.1093/geroni/igz038.3320

**Published:** 2019-11-08

**Authors:** W Quin Yow, Hui-Ching Chen, Tharshini Lokanathan

**Affiliations:** Singapore University of Technology & Design, Singapore, Singapore

## Abstract

Dementia, a prevalent ageing disease, affects both the higher brain function and motor function, particularly finger movements (Chan, Haber, Drew, & Park, 2014). Task-based finger-tapping speed on a touchscreen device has been used as an assessment criterion to identify patients with deteriorating cognitive abilities (Gualtieri & Johnson, 2005; Cipriani, Bianchetti, & Trabucchi, 2006). As part of a larger project, we designed a computerized cognition intervention program and examined whether the intervention program would improve the finger-tapping speed of the dementia vis-à-vis the cognitively-healthy elderly. Ten mild-to-moderate dementia elderly (aged 83± 5.6) and 8 cognitively healthy elderly (aged 78±6.1) participated in a computerized intervention program where they played cognitive games on touch-screen tablet for about 30-45 minutes per session over two weeks. Participants’ touch interaction data over six sessions were collected and analyzed. Using a linear mixed-effect model for analysis, we found that in the 1st session, the touch performance of the dementia elderly was significantly worse than that of the cognitively-healthy elderly (b=-0.172, Z=-2.311, p<.05). By the 6th session, the dementia elderly had significantly improved their touch performance (b=-0.171, Z= -8.042, p<.001) such that their touch performance was now comparable to the cognitively-healthy elderly (b=-0.064, Z=-0.874, p=.393). Overall, our preliminary results suggested that after participating in 6 sessions of our computerized cognitive intervention program, the dementia elderly showed significant improvement in their fine motor movement as measured by their finger-tapping speed. The improved finger-tapping speed serves as a first step toward slowing down the cognitive decline of the dementia elderly.

